# Estimated glomerular filtration rate and cardiometabolic risk factors in a longitudinal cohort of children

**DOI:** 10.1038/s41598-021-91162-x

**Published:** 2021-06-03

**Authors:** Sílvia Xargay-Torrent, Elsa Puerto-Carranza, Irene Marcelo, Berta Mas-Parés, Ariadna Gómez-Vilarrubla, Jose-Maria Martínez-Calcerrada, Francis de Zegher, Lourdes Ibáñez, Abel López-Bermejo, Judit Bassols

**Affiliations:** 1 Pediatric Endocrinology Research Group, Girona Biomedical Research Institute (IDIBGI), 17190 Salt, Spain; 2 Pediatrics, Dr. Trueta University Hospital, 17007 Girona, Spain; 3 Maternal-Fetal Metabolic Research Group, Girona Biomedical Research Institute (IDIBGI), Parc Hospitalari Martí I Julià, Edifici M2, 17190 Salt, Spain; 4 Department of Development and Regeneration, University of Leuven, 3000 Leuven, Belgium; 5 Endocrinology, Pediatric Research Institute, Sant Joan de Déu Children’s Hospital, 08950 Esplugues, Barcelona Spain; 6 Spanish Biomedical Research Centre in Diabetes and Associated Metabolic Disorders, CIBERDEM, ISCIII, 28029 Madrid, Spain; 7 Department of Medical Sciences, Faculty of Medicine, University of Girona, 17003 Girona, Spain

**Keywords:** Paediatric research, Endocrine system and metabolic diseases

## Abstract

Associations between glomerular filtration rate (GFR) and cardiometabolic risk factors have been reported in adult and pediatric patients with renal disease. We aimed to assess the relationship between the estimated GFR (eGFR) and cardiometabolic risk factors in apparently healthy children. A longitudinal study in 401 asymptomatic Caucasian children (mean age 8 years) followed up after 4 years (mean age 12 years). GFR was estimated using the pediatric form of the FAS-equation. Children were classified at baseline according to their obesity status (normal weight and overweight) and according to eGFR levels (lower, average, and higher). The association of eGFR with anthropometric data [body mass index (BMI) and waist], blood pressure [systolic (SBP) and diastolic (DBP)], metabolic parameters [glucose, insulin resistance (HOMA-IR) and serum lipids], and renal ultrasonography measurements were assessed at baseline and follow-up. Baseline eGFR associated with several cardiometabolic risk factors at follow-up including higher waist, SBP, HOMA-IR, and kidney size (all *p* < 0.0001) in both normal weight and overweight children. In multivariate analysis, baseline eGFR was independently associated with follow-up HOMA-IR and SBP in both normal weight and overweight subjects (model *R*^2^: 0.188–0.444), and with follow-up BMI and waist in overweight subjects (model *R*^2^: 0.367–0.477). Moreover, children with higher filtration rates at baseline showed higher waist, SBP, DBP, HOMA-IR and renal size both at baseline and follow-up. eGFR is related to insulin resistance, blood pressure and adiposity measures in school-age children. eGFR may help to profile the cardiometabolic risk of children.

## Introduction

The increasing prevalence of obesity in children has emerged as one of the major public health problems in recent years^1^. Prevention is a worldwide challenge to be addressed to restrain this condition of epidemic dimensions. The metabolic syndrome that comprises several comorbidities [such as type 2 diabetes, hypertension, and cardiovascular (CV) disease] had rather been associated with obesity in adulthood, however, it may also coexist in children and adolescents with obesity today^2^. Moreover, several studies have pointed out the alarming increase in the prevalence of CV risk factors not only in children with obesity^3^ but also in the general pediatric population ^4,5^.

Growing interest is focused on the estimated glomerular filtration rate (eGFR) as this measure proved to be a useful tool for estimating renal function and an accurate indicator of CV risk^6^. In adults, impaired eGFR has been related to several pathological conditions, including diabetes^7^, hypertension^8^, and obesity^9^, and it was shown to be an independent predictor of end-stage renal disease and cardiovascular disease both in adult subjects with and without diabetes^10^.

Limited information is available on the relationship between eGFR and cardiometabolic risk in the pediatric population. The majority of these studies are primarily disease-related (i.e. chronic kidney disease, diabetes, obesity) and cross-sectionally designed^11–15^. In the present study, we aimed to study the relationship between eGFR and several cardiometabolic risk parameters in a pediatric population of school-age children both in cross-sectional and longitudinal analyses.

## Subjects and methods

### Study population and ethics

The study cohort comprised a total of 849 apparently healthy school-age children [5–12 years (408 girls and 441 boys)] recruited in a prospective longitudinal study of obesity and cardiovascular risk factors among those seen in primary care settings in Girona and Figueres between 2009 and 2015, regions in northeastern Spain. A total of 401 families consented to reassess their child after 4 years (211 girls, 190 boys). Children with major congenital anomalies (abnormal liver, kidney, or thyroid functions), health conditions (evidence of chronic or acute illness), or prolonged use of medication in the month preceding potential enrolment were excluded from the study. The study was approved by the Institutional Review Board of Dr. Josep Trueta Hospital and was carried out according to The Code of Ethics of the World Medical Association (Declaration of Helsinki). Informed written consent was obtained from all the parents.

### Clinical assessments

Subjects were weighed on a calibrated scale and the height was measured with a Harpenden stadiometer. Body-mass index (BMI) was calculated as weight (in kg) divided by the square of height (in meters). Age- and sex-adjusted standard deviation scores (SDS) for BMI were calculated using regional normative data^16^. The study subjects were grouped according to their BMI-SDS into normal weight (< 1 SDS) and overweight (≥ 1 SDS)^17^. Waist circumference was measured in the supine position at the umbilical level with a metric tape. Hip circumference was measured at the widest part, at the level of the greater trochanters. Systolic and diastolic blood pressure (SBP and DBP) were measured using an electronic sphygmomanometer (Dinamap Pro 100, GE Healthcare, Chalfont St. Giles, UK) after a 10-min-rest on the right arm for 3 consecutive times with the child in the supine position. The average of the two most similar measurements was used in the analysis. The percentage of hypertensive subjects was determined as previously described^18^. Puberty was evaluated according to Tanner criteria. Renal size (length, depth, and width) was measured by high-resolution ultrasonography (MyLabTM25, Esaote, Firenze, Italy) as previously reported^19^. The renal size was measured in both kidneys with the subjects placed in left lateral supine position. Given that the right kidney was more easily assessed, and therefore such measurements were more reproducible, we only show data of the right kidney. Averages of three measurements for each parameter were used in the study and renal volume was calculated using the following formula [length * depth * width * 0.523]^20^. Results for renal length and volume were available for all patients. A pediatric nurse took all measurements except for ultrasonography, which was performed by a specialized technician.

### Laboratory variables

Blood samples were obtained in the morning after an overnight fast. Serum glucose was measured by the hexokinase method. Insulin was measured by immunochemiluminiscence (IMMULITE 2000, Diagnostic Products, Los Angeles, CA, USA). The lower detection limit was 0.4 mIU/L and intra- and inter-assay CVs were < 10%. Insulin resistance was estimated by the homeostasis model assessment [HOMA-IR = fasting insulin (mg/dl) × fasting glucose (mM)/405]. Total serum triacylglycerol (TG) was measured by glycerol-phosphate oxidase method and HDL cholesterol by a homogeneous method of selective detergent with accelerator (ARCHITECT, Abbott Laboratories, Abbott Park, IL, USA). Lower detection limits were 5.0 mg/dL and 2.5 mg/dL, respectively; and intra- and inter-assay CVs < 5%. Serum creatinine (Scr) was measured by the enzymatic method (COBAS 702, Roche Diagnostics, IN). eGFR was calculated by the pediatric form of the FAS-equation: [eGFR = 107.3/(Scr/Q)] where Q = 0.0270 × Age + 0.2329^21^.

We categorized eGFR using the following clinically relevant cut-points established by the National Kidney Foundation’s Kidney Disease Outcomes Quality Initiative (KDOQI): lower filtration (< 90 mL/min/1.73 m^2^), average filtration (90–119 mL/min/1.73 m^2^), and higher filtration rate (≥ 120 mL/min/1.73 m^2^).

### Statistics

The Statistical Package for Social Sciences SPSS 22.0 (IBM Corp, Amonk, NY, USA) was used to perform statistical analyses. In a bilateral contrast, accepting an alpha risk of 0.05, our study with 401 subjects (283 normal weight and 118 overweight) has a 90% power to detect a significant Pearson correlation coefficient of at least 0.25 between eGFR and cardiometabolic variables (GRANMO, IMIM, version 7.12).

Results are presented as mean ± standard deviation (SD), or *n* (%). Differences among groups were assessed by Student’s *t*-test (normal weight vs overweight) and one-way ANOVA (eGFR categories). The ANOVA results were corrected for multiple comparisons using the Bonferroni test. Distribution of categories were studied by chi-square. Associations between continuous variables were studied by Pearson’s method; multiple regression analyses were used to study independent associations between eGFR at baseline and endocrine-metabolic variables at follow-up, adjusting for potentially confounding factors. Variables without normal distribution were mathematically transformed to improve symmetry. The relative risk of having higher BMI, SBP, and HOMA-IR at follow-up based on baseline eGFR values was measured using Odds Ratio (OR). *P* values < 0.05 were considered statistically significant.

## Results

Our study included 401 subjects from a cohort of school-age children, who were initially assessed at a mean age of 8 years and reassessed after 4 years of follow-up. Children were classified at baseline into children with normal weight (BMI-SDS < 1) and with overweight (BMI-SDS ≥ 1). Supplementary Table 1 shows the clinical, laboratory, and ultrasonography assessments in the studied subjects both at baseline and follow-up in the whole sample and in the study subgroups (normal weight and overweight). The comparison between baseline and follow-up data in normal weight and overweight groups is shown in Table 1.

**Table 1:** Comparison between baseline and follow-up data in normal weight and overweight groups.

	Normal weight			Overweight		
	Baseline	Follow-up	*p* value	Baseline	Follow-up	*p* value
BMI (kg/m^2^)	16.4 ± 2.1	18.7 ± 3.1	< 0.0001	23.8 ± 3.0	27.5 ± 4.6	< 0.0001
BMI-SDS	− 0.3 ± 0.6	− 0.2 ± 0.8	0.01	2.0 ± 0.8	2.0 ± 1.2	NS
Height (cm)	126.4 ± 12.3	149.3 ± 13.2	< 0.0001	137.5 ± 13.1	160.1 ± 11.9	< 0.0001
Height-SDS	0.1 ± 1.1	0.2 ± 1.0	NS	1.0 ± 1.0	0.8 ± 0.9	0.001
Weight (kg)	26.8 ± 8.2	42.8 ± 13.0	< 0.0001	46.3 ± 13.3	71.0 ± 16.8	< 0.0001
Weight-SDS	− 0.1 ± 0.8	− 0.1 ± 0.8	NS	2.1 ± 0.9	2.1 ± 1.1	NS
Waist (cm)	55.8 ± 7.8	67.1 ± 9.6	< 0.0001	75.6 ± 10.1	88.8 ± 10.6	< 0.0001
SBP (mmHg)	102.8 ± 9.3	106.2 ± 11.6	< 0.0001	109.6 ± 11.8	118.6 ± 12.1	< 0.0001
DBP (mmHg)	58.5 ± 6.9	59.2 ± 7.2	NS	62.3 ± 7.8	63.3 ± 8.7	NS
Glucose (mg/dL)	85.6 ± 6.4	86.8 ± 6.6	0.003	87.1 ± 6.4	86.7 ± 6.8	NS
Creatinine (mg/dL)	0.50 ± 0.09	0.58 ± 0.10	< 0.0001	0.51 ± 0.07	0.65 ± 0.11	< 0.0001
Insulin (µU/mL)	2.9 ± 3.0	6.9 ± 4.2	< 0.0001	7.9 ± 6.3	13.4 ± 7.8	< 0.0001
HOMA-IR	0.6 ± 0.6	1.4 ± 0.9	< 0.0001	1.7 ± 1.3	2.9 ± 1.7	< 0.0001
HDL-cholesterol (mg/dL)	60.6 ± 13.7	60.6 ± 14.9	NS	49.3 ± 10.5	49.2 ± 11.7	NS
LDL-cholesterol (mg/dL)	91.9 ± 24.3	79.4 ± 22.4	< 0.0001	96.9 ± 22.8	85.9 ± 22.7	< 0.0001
Total cholesterol (mg/dL)	169.3 ± 29.8	159.7 ± 30.7	< 0.0001	162.5 ± 24.9	153.0 ± 24.0	< 0.0001
Triacylglycerol (mg/dL)	53.4 ± 22.3	57.2 ± 22.2	0.007	73.3 ± 37.0	82.2 ± 40.7	0.03
eGFR (mL/min/1.73 m^2^)	95.45 ± 18.1	101.8 ± 16.2	< 0.0001	99.4 ± 17.1	97.9 ± 14.8	NS
Renal length (cm)	8.4 ± 0.8	9.5 ± 0.9	< 0.0001	9.2 ± 1.0	10.5 ± 1.1	< 0.0001
Renal volume (cm^3^)	68.2 ± 18.5	91.8 ± 30.6	< 0.0001	88.5 ± 25.6	126.7 ± 39.9	< 0.0001

*BMI* body mass index, *SBP* systolic blood pressure, *DBP* diastolic blood pressure, *eGFR* estimated glomerular filtration rate, *HOMA-IR* homeostatic model assessment of insulin resistance, *NS* non-significant.

*P *value < 0.05 by paired *t *test analysis.

### Association between eGFR and cardiometabolic risk factors

**Figure 1:**
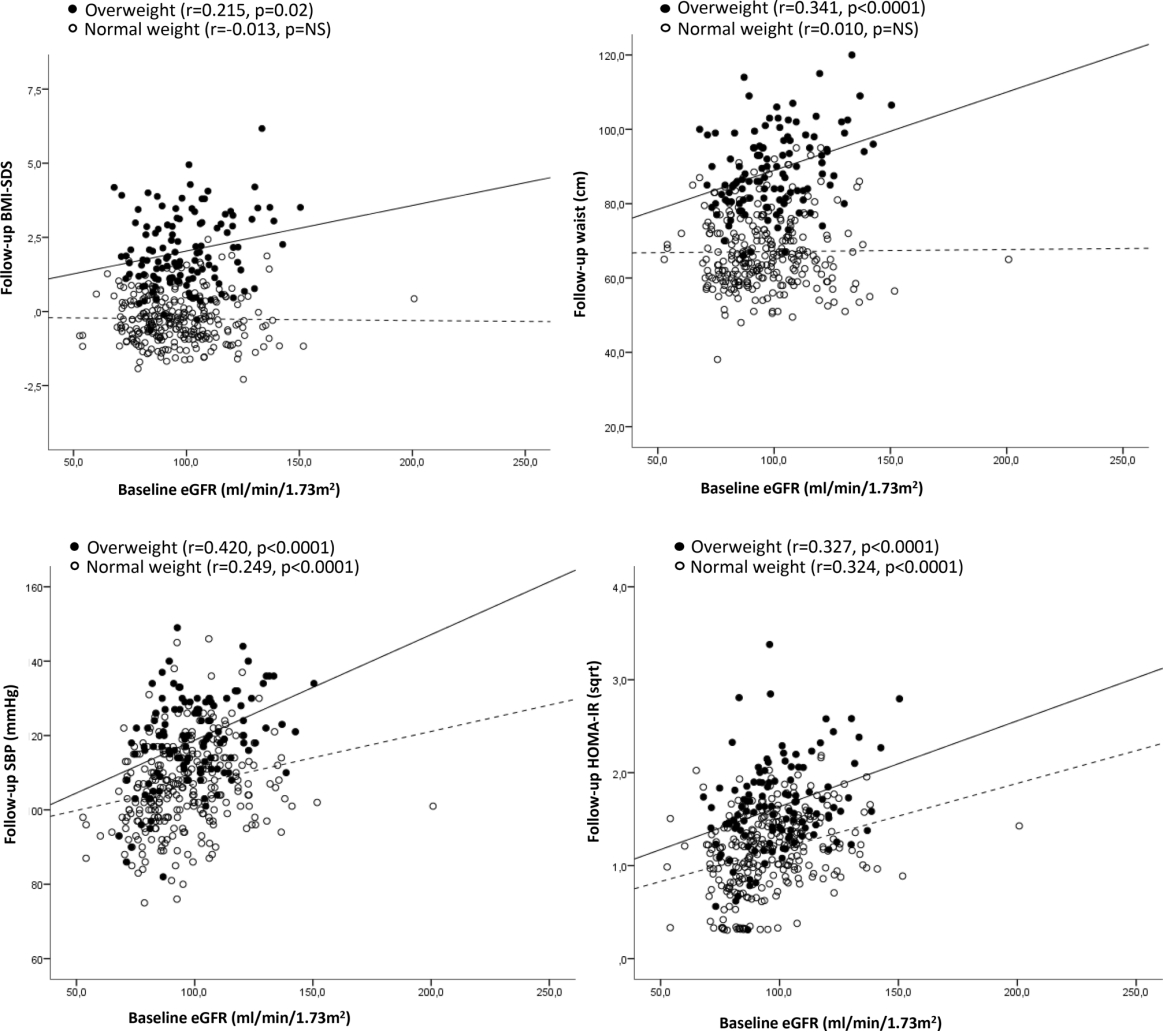
Correlation graphs of eGFR at baseline with selected variables at follow-up (n = 401). White dots and dotted line depict children with normal weight (BMI-SDS < 1) and black dots and solid line, children with overweight (BMI-SDS ≥ 1).

Baseline eGFR showed positive associations with several anthropometric and cardiometabolic risk parameters at follow-up including higher waist circumference, SBP, HOMA-IR, and kidney length and volume (all r between 0.315 and 0.450, *p* < 0.0001; Table 2). These associations were stronger in children with overweight (Table 2; Fig. 1).

**Table 2:** Pearson correlation analyses of eGFR at baseline with selected variables at follow-up.

Baseline eGFR	All (N = 401)		Normal weight (N = 283)		Overweight (N = 118)	
Follow-up	r	*p* value	r	*p* value	r	*p* value
Age (years)	**0.334**	< **0.0001**	0.239	< 0.0001	**0.450**	< **0.0001**
BMI-SDS	0.120	0.01	− 0.013	NS	0.215	0.02
Height-SDS	0.006	NS	− 0.048	NS	0.010	NS
Weight-SDS	0.112	0.02	− 0.035	NS	0.224	0.01
Waist (cm)	0.151	0.003	0.010	NS	**0.341**	< **0.0001**
SBP (mmHg)	**0.315**	< **0.0001**	0.249	< 0.0001	**0.420**	< **0.0001**
DBP (mmHg)	0.219	< 0.0001	0.221	< 0.0001	0.141	NS
Glucose (mg/dL)	− 0.084	NS	− 0.100	NS	− 0.056	NS
Creatinine (mg/dl)	− 0.205	< 0.0001	− **0.314**	< **0.0001**	− 0.123	NS
Insulin (µU/mL)	**0.354**	< **0.0001**	**0.341**	< **0.0001**	**0.346**	< **0.0001**
HOMA-IR	**0.341**	< **0.0001**	**0.324**	< **0.0001**	**0.327**	< **0.0001**
HDL-cholesterol (mg/dL)	0.056	NS	0.135	0.02	− 0.032	NS
LDL-cholesterol (mg/dL)	0.091	NS	0.075	NS	0.138	NS
Total cholesterol (mg/dL)	− 0.110	0.024	− 0.119	0.04	− 0.026	NS
Triacylglycerol (mg/dL)	0.067	NS	0.024	NS	0.037	NS
Renal length (cm)	**0.317**	< **0.0001**	0.205	0.001	**0.463**	< **0.0001**
Renal volume (cm^3^)	**0.372**	**<0.0001**	0.259	<0.0001	**0.536**	**<0.0001**

*BMI* body mass index, *SBP* systolic blood pressure, *DBP* diastolic blood pressure, *eGFR* estimated glomerular filtration rate, *HOMA-IR* homeostatic model assessment of insulin resistance, *NS* non-significant.

Moderate associations (Pearson’s r > 0.3) are shown in bold.

In multivariate analyses adjusting for confounding variables (including sex, age, puberty and BMI at baseline), baseline eGFR was independently associated with follow-up BMI (β = 0.184, *p* = 0.01, model R^2^ = 0.477) and waist (β = 0.198, *p* = 0.02, model R^2^ = 0.367) in children with overweight and with follow-up SBP (normal weight: β = 0.121, *p* = 0.01, model R^2^ = 0.444; overweight: β = 0.199, *p* = 0.02, model R^2^ = 0.377) and follow-up HOMA-IR (normal weight: β = 0.217, *p* < 0.0001, model R^2^ = 0.257; overweight: β = 0.244, *p* = 0.01, model R^2^ = 0.188) in both children with normal weight and overweight (Table 3).

**Table 3:** Multivariate linear models of baseline eGFR as the independent variable and selected variables at follow-up.

	All subjects		Normal weight subjects		Overweight subjects	
	Beta	Sig	Beta	Sig	Beta	Sig
**Follow-up BMI**						
**Baseline eGFR**	–	–	–	–	0.184	0.01
Baseline BMI	0.889	< 0.0001	0.750	–	0.722	< 0.001
R^2^		0.791		0.596		0.477
Non-predictive variables: age, sex and puberty at baseline						
**Follow-up waist**						
**Baseline eGFR**	–	–	–	–	0.198	0.02
Baseline BMI	0.823	< 0.0001	0.646	< 0.0001	0.556	< 0.0001
Baseline age	–	–	0.115	0.02	–	–
R^2^		0.715		0.533		0.367
Non-predictive variables: sex and puberty at baseline						
**Follow-up SBP**						
**Baseline eGFR**	0.120	0.001	0.121	0.01	0.199	0.02
Baseline BMI	0.264	< 0.0001	0.149	0.006	–	–
Baseline SBP	0.329	< 0.0001	0.379	< 0.0001	0.227	0.002
Baseline age	0.274	< 0.0001	0.321	< 0.0001	0.260	0.02
R^2^		0.513		0.444		0.377
Non-predictive variables: sex and puberty at baseline						
**Follow-up HOMA-IR**						
**Baseline eGFR**	0.180	< 0.0001	0.217	< 0.0001	0.244	0.01
Baseline BMI	0.356	< 0.0001	0.157	0.01	–	–
Baseline HOMA-IR	0.216	< 0.0001	0.181	0.004	0.260	0.01
Baseline age	–	–	0.189	0.005	–	–
Sex	0.125	0.003	0.161	0.003	–	–
R^2^		0.356		0.257		0.188
Non-predictive variables: puberty at baseline						

To show the potential clinical use of eGFR in predicting cardiovascular risk, we calculated the relative risk of having higher BMI, SBP, and HOMA-IR at follow-up based on baseline eGFR values. The results showed that children with higher baseline eGFR levels (*p* > 50 eGFR) were at increased risk of being overweight (BMI-SDS ≥ 1) [Odds Ratio (OR) 1.23; 95% Confidence Interval (CI) 1.01–1.49] and of having higher SBP (*p* > 50) [OR 1.76; CI 1.42–2.17] and higher HOMA-IR (*p* > 50) [OR 1.75; CI 1.41–2.16] at follow-up.

### CV risk factors according to eGFR categories

We also categorized the population according to relevant cut-points of baseline eGFR (Table 4). Statistically significant differences across categories were observed in several baseline parameters including age, waist, SBP, DBP, insulin, HOMA-IR, and renal size (all *p* ≤ 0.01, Table 4). At follow-up, SBP, DBP, insulin, HOMA-IR, and renal size differed across baseline eGFR categories after adjustment for confounding variables (sex and baseline age, BMI, and puberty) (all *p* < 0.0001).

**Table 4:** Clinical, laboratory, and ultrasonography assessments at baseline and follow-up according to baseline eGFR categories.

	Lower filtration (< 90 mL/min/1.73 m^2^)	Average filtration (90–119 mL/min/1.73 m^2^)	Higher filtration (≥ 120 mL/min/1.73 m^2^)	*p* value
	N = 160	N = 197	N = 44	
**Baseline**				
eGFR (mL/min/1.73 m^2^)	80.2 ± 7.0^bc^	102.3 ± 7.7^ac^	130.8 ± 13.4^ab^	< 0.0001
Age (years)	7 ± 1^bc^	8 ± 2^a^	8 ± 2^a^	< 0.0001
Sex (female %)	49	54	57	NS
Puberty (%)	6	12	18	0.02
Overweight/obesity (%)	24	31	39	NS
BMI-SDS	0.2 ± 1.2	0.3 ± 1.3	0.6 ± 1.4	NS
Height-SDS	0.3 ± 1.1	0.5 ± 1.1	0.3 ± 1.4	NS
Weight-SDS	0.3 ± 1.2	0.5 ± 1.3	0.7 ± 1.6	NS
Waist (cm)	58.2 ± 10.1^bc^	63.3 ± 12.6^a^	66.8 ± 15.5^a^	< **0.0001**
SBP (mmHg)	102.9 ± 8.9^c^	105.5 ± 11.0	108.1 ± 12.6^a^	**0.007**
DBP (mmHg)	58.5 ± 7.3^c^	60.0 ± 7.5	62.0 ± 6.7^a^	**0.01**
Hypertension (%)	4	5	7	NS
Creatinine (mg/dL)	0.57 ± 0.07^bc^	0.47 ± 0.06^ac^	0.38 ± 0.06^ab^	< **0.0001**
Glucose (mg/dL)	85.4 ± 6.2	86.4 ± 6.8	86.4 ± 6.0	NS
Insulin (µU/mL)	3.3 ± 3.8^bc^	4.7 ± 4.6^ac^	6.8 ± 7.5^ab^	< 0.0001
HOMA-IR	0.7 ± 0.8^bc^	1.0 ± 0.9^ac^	1.4 ± 1.5^ab^	< 0.0001
HDL-cholesterol (mg/dL)	56.7 ± 11.8	58.4 ± 14.7	58.0 ± 15.3	NS
LDL-cholesterol (mg/dL)	84.7 ± 19.1^c^	94.1 ± 23.1	99.3 ± 27.8^a^	0.03
Total cholesterol (mg/dL)	169.7 ± 29.6	164.6 ± 26.8	170.5 ± 31.9	NS
Triacylglycerol (mg/dL)	58.4 ± 23.8	59.0 ± 31.3	63.8 ± 34.2	NS
Renal length (cm)	8.5 ± 0.8^c^	8.6 ± 0.9^c^	9.1 ± 1.2^ab^	**0.005**
Renal volume (cm^3^)	66.6 ± 18.7^bc^	76.3 ± 20.9^ac^	87.9 ± 34.4^ab^	< **0.0001**
**Follow-up**				
eGFR (mL/min/1.73 m^2^)	93.1 ± 11.3^bc^	103.2 ± 15.0^ac^	117.7 ± 17.5^ab^	< 0.0001
Age (years)	11 ± 2^bc^	12 ± 2^a^	13 ± 2^a^	< 0.0001
Puberty (%)	56	65	74	NS
Overweight/obesity (%)	22	32	36	NS
BMI-SDS	0.2 ± 1.2	0.4 ± 1.3	0.8 ± 1.8	NS
Height-SDS	0.3 ± 1.1	0.4 ± 1.0	0.1 ± 1.0	NS
Weight-SDS	0.3 ± 1.2	0.6 ± 1.3	0.7 ± 1.8	NS
Waist (cm)	70.8 ± 12.3^bc^	74.7 ± 13.9^a^	77.0 ± 18.4^a^	0.007
SBP (mmHg)	105.3 ± 11.8^bc^	112.4 ± 12.6^a^	115.2 ± 13.9^a^	< **0.0001**
DBP (mmHg)	58.0 ± 7.8^bc^	62.1 ± 7.5^a^	61.5 ± 8.1^a^	< **0.0001**
Hypertension (%)	1	4	2	NS
Glucose (mg/dL)	86.9 ± 5.8	87.0 ± 7.0	85.3 ± 7.6	NS
Creatinine (mg/dL)	0.61 ± 0.08^c^	0.60 ± 0.12^c^	0.54 ± 0.11^ab^	< 0.0001
Insulin (µU/mL)	6.3 ± 4.9^bc^	10.2 ± 6.4^a^	11.5 ± 7.2^a^	< **0.0001**
HOMA-IR	1.3 ± 1.1^bc^	2.2 ± 1.4^a^	2.4 ± 1.6^a^	< **0.0001**
HDL-cholesterol (mg/dL)	57.3 ± 12.5	57.2 ± 16.0	57.8 ± 18.3	NS
LDL-cholesterol (mg/dL)	78.7 ± 25.3	81.1 ± 22.2	87.1 ± 24.5	NS
Total cholesterol (mg/dL)	163.8 ± 28.4^b^	152.6 ± 28.8^a^	158.6 ± 28.4	0.001
Triacylglycerol (mg/dL)	60.2 ± 26.1^c^	67.4 ± 34.3	67.6 ± 30.6	NS
Renal length (cm)	9.4 ± 0.8^bc^	10.0 ± 1.0^a^	10.3 ± 1.4^a^	< **0.0001**
Renal volume (cm^3^)	86.1 ± 26.2 ^bc^	110.2 ± 35.6 ^a^	124.2 ± 50.4 ^a^	< **0.0001**

Data is presented as mean ± standard deviation (SD), or n (%).

*BMI* body mass index, *SBP* systolic blood pressure, *DBP* diastolic blood pressure, *eGFR* glomerular filtration rate, *HOMA-IR* homeostatic model assessment of insulin resistance, *NS* non-significant. Continuous variables are compared with the ANOVA and the Bonferroni test, and proportions are compared with the chi-square.

^a^*p* < 0.05 compared to lower filtration.

^b^*p* < 0.05 compared to average filtration.

^c^*p* < 0.05 compared to higher filtration.

Significant after adjustment for pubertal stage, sex, age, and BMI in univariate general linear models are shown in bold.

## Discussion

In this study, we assessed the relationship between eGFR and cardiometabolic risk factors in school-age children both cross-sectionally and longitudinally. Our results indicated that baseline eGFR was associated with several cardiometabolic parameters at baseline and follow-up in both children with normal weight and overweight. Moreover, baseline eGFR was independently related to insulin resistance, blood pressure, and obesity measures at follow-up.

Metabolism and renal function seem to have a bi-directional relationship. On one hand, it is well known that diabetes is the most common cause of kidney disease. A large percentage of patients who progress to end-stage renal disease have diabetes. Other underlying conditions in patients with end-stage renal disease include hypertension, glomerulonephritis, and cystic kidney^22^. eGFR, on the other hand, has been validated as a useful approach to evaluate cardiovascular risk. This measure has been especially useful in clinical adult populations, in which the assessment of eGFR was already shown (in individuals with chronic kidney disease, diabetes, and hypertension) to more accurately predict cardiovascular events compared to traditional risk factors^23^. Regarding studies in children and adolescents, it has been shown that excess body weight is positively correlated with renal disease. Furthermore, several authors have demonstrated that eGFR was a good tool to identify cardiovascular risk among healthy pediatric populations with a high prevalence of obesity^11,12,14^.

Our results are in agreement with the previous literature and showed that eGFR may be a potential predictor of several cardiometabolic risk factors including HOMA-IR, blood pressure, and obesity measures (BMI and waist) in apparently healthy children after 4 years of follow-up. These associations were observed in all the study population and in subgroups according to baseline obesity status (normal weight and overweight). Independent associations with follow-up HOMA-IR and SBP were observed in both groups. In this sense, the PREVEND Study Group, reported independent associations between central body fat distribution and renal function in adult individuals with normal weight and overweight/obesity^24^, suggesting that the pattern of fat distribution, rather than obesity itself, seems to be most associated with renal function.

Insulin resistance, a key risk factor for type 2 diabetes has also been associated with abnormally high glomerular filtration rate in adults^25^. It has been suggested that insulin, through its effects on renal hemodynamics, may influence the glomerular filtration rate^26–28^. Importantly, amelioration of glomerular hyperfiltration in type 2 diabetes is associated with both improved insulin sensitivity and a slower decline in renal function over time^29^. Ricotti et al. have recently shown that young patients with an eGFR greater than 1 SD had higher HOMA-IR and insulin during oral glucose tolerance test (OGTT)^12^. Our results are in line with these observations.

Hypertension, which is common among people with obesity and insulin resistance, has also been linked to renal function. Glomerular hyperfiltration increases with increasing stages of prediabetes and prehypertension^30^. Type 1 diabetes patients with elevated GFR values show a faster decline in renal function than those with impaired or reduced GFR, and more rapid loss of kidney function was related to long-term hypertension^31^. Association between renal function and hypertension has also been reported in patients with obesity secondary to glomerular capillary endothelial cell injury^32^. In agreement with our results, several studies highlight the association between blood pressure and renal function in healthy children and children with obesity^11–14^.

We also categorized the population according to relevant cut-points of baseline eGFR and observed that children with higher filtration (≥ 120 mL/min/1.73 m^2^), showed higher waist, SBP, DBP, insulin, HOMA-IR, and renal size compared to those with lower and average filtration at both baseline and follow-up. On the contrary, a recent study by Di Bonito (2021)^33^ showed that, in young overweight subjects, mildly reduced estimated glomerular filtration rate (MRGFR) (eGFR > 60 and < 90 mL/min/1.73 m^2^) was associated with an abnormal cardiometabolic risk profile, including higher BMI and SBP. The divergence of these results with ours could be explained by the obesity status of the large study population in the Di Bonito study, as the children with lower eGFR in our study were not predominantly overweight subjects. Additionally, we cannot rule out the possibility that a U-shape relationship exists between glomerular filtration rate and cardiovascular risk factors, with subjects in both ends of the spectrum of the eGFR showing increased risk for cardiovascular disease, depending also on age and body weight^34^.

The major strength of this study is its longitudinal design, being possible to explore a contributing relationship between eGFR and cardiometabolic parameters over time. Furthermore, this study comprises a large sample of a well-characterized pediatric group of children with normal weight and overweight. Among the limitations, we recognize that the correlations observed in our results are moderate (Pearson’s r between 0.3 and 0.7), which may limit the significance of our results; however, the longitudinal design of the study is of clinical relevance. Among the different methods used to measure the glomerular filtration rate, the GFR equations (eGFR) are the most often used in clinical practice because they only required knowledge of the serum creatinine or cystatin C levels and demographic data. However, it should be noted that most of the eGFR equations (Schwartz and Pottel) may erroneously report “hyperfiltration” when serum creatinine is low. To explore whether baseline eGFR could be an independent predictor of cardiometabolic risk, we have performed multiple regression analyses adjusting for sex, age, puberty and BMI at baseline; however, we recognize that additional factors may be involved in these associations that were not taken into account in our study. Finally, when interpreting the results of our study in terms of risk for cardiometabolic abnormalities, it should be pointed out that our study did not deal with pathological conditions; indeed, all children were healthy and therefore, at present, their cardiometabolic parameters were within the normal range; however, we cannot exclude that these parameters would be out of range in later life in those children with higher eGFR.

In conclusion, we showed that eGFR at baseline was associated with HOMA-IR, SBP, and obesity parameters at baseline and follow-up, in both normal weight children and overweight children, independently of other well-established risk factors. Moreover, our analysis showed that children with higher baseline eGFR levels were at increased risk of being overweight and having higher SBP and higher HOMA-IR at follow-up. Thus, the present findings led us to suggest monitoring eGFR in the early prevention of insulin resistance, obesity, and cardiovascular risk in children.

## Supplementary Information

Supplementary Information.

## Data Availability

The datasets generated during and/or analyzed during the current study are not publicly available but are available from the corresponding author on reasonable request.
