# Supplementary material for: Estimated glomerular filtration rate and cardiometabolic risk factors in a longitudinal cohort of children
Source: Sci Rep. 2021 Jun 3;11:11702. doi: 10.1038/s41598-021-91162-x (PMC8175594; doi:10.1038/s41598-021-91162-x)
Supplement: Supplementary file 1 — Supplementary Information. [file 41598_2021_91162_MOESM1_ESM.docx]

**Supplementary Table 1.** Clinical, laboratory, and ultrasonography assessments

|  | **All** | **Normal weight** | **Overweight** | **P-value (normal weight**  **vs overweight)** |
| --- | --- | --- | --- | --- |
| **BASELINE** | **N=401** | **N=283** | **N=118** |  |
| Age (year) | 7 ± 2 | 7 ± 2 | 8 ± 2 | <0.0001 |
| Sex (female %) | 52 | 51 | 54 | Ns |
| Puberty (%) | 10 | 6 | 20 | <0.0001 |
| BMI (kg/m2) | 18.6 ± 4.1 | 16.4 ± 2.1 | 23.8 ± 3.0 | <0.0001 |
| BMI-SDS | 0.3 ± 1.3 | -0.3 ± 0.6 | 2.0 ± 0.8 | <0.0001 |
| Height (cm) | 129.7 ± 13.5 | 126.4 ± 12.3 | 137.5 ± 13.1 | <0.0001 |
| Height-SDS | 0.4 ± 1.1 | 0.1 ± 1.1 | 1.0 ± 1.0 | <0.0001 |
| Weight (Kg) | 32.5 ± 13.3 | 26.8 ± 8.2 | 46.3 ± 13.3 | <0.0001 |
| Weight-SDS | 0.5 ± 1.3 | -0.1 ± 0.8 | 2.1 ± 0.9 | <0.0001 |
| Waist (cm) | 61.6 ± 12.4 | 55.8 ± 7.8 | 75.6 ± 10.1 | <0.0001 |
| SBP (mmHg) | 104.7 ± 10.5 | 102.8 ± 9.3 | 109.6 ± 11.8 | <0.0001 |
| DBP (mmHg) | 59.6 ± 7.4 | 58.5 ± 6.9 | 62.3 ± 7.8 | <0.0001 |
| Hypertension (%) | 4 | 3 | 8 | 0.01 |
| Glucose (mg/dl) | 86.0 ± 6.5 | 85.6 ± 6.4 | 87.1 ± 6.4 | 0.02 |
| Creatinine (mg/dl) | 0.50 ± 0.09 | 0.50 ± 0.09 | 0.51 ± 0.07 | Ns |
| Insulin (uU/ml) | 4.4 ± 4.8 | 2.9 ± 3.0 | 7.9 ± 6.3 | <0.0001 |
| HOMA-IR | 0.9 ± 1.0 | 0.6 ± 0.6 | 1.7 ± 1.3 | <0.0001 |
| HDL-cholesterol (mg/dl) | 57.7 ± 13.7 | 60.6 ± 13.7 | 49.3 ± 10.5 | <0.0001 |
| LDL-cholesterol (mg/dl) | 93.8 ± 23.8 | 91.9 ± 24.3 | 96.9 ± 22.8 | Ns |
| Total cholesterol (mg/dl) | 167.3 ± 28.6 | 169.3 ± 29.8 | 162.5 ± 24.9 | 0.03 |
| Triacylglycerol (mg/dl) | 59.3 ± 28.8 | 53.4 ± 22.3 | 73.3 ± 37.0 | <0.0001 |
| eGFR (ml/min/1.73m^2^) | 96.6 ± 17.8 | 95.45 ± 18.1 | 99.4 ± 17.1 | 0.04 |
| Renal length (cm) | 8.6 ± 0.9 | 8.4 ± 0.8 | 9.2 ± 1.0 | <0.0001 |
| Renal volume (cm^3^) | 74.4 ± 23.3 | 68.2 ± 18.5 | 88.5 ± 25.6 | <0.0001 |
| **FOLLOW-UP** |  |  |  |  |
| Age (year) | 12 ± 2 | 12 ± 2 | 13 + 2 | <0.0001 |
| Puberty (%) | 63 | 54 | 80 | <0.0001 |
| BMI (kg/m2) | 21.3 ± 5.4 | 18.7 ± 3.1 | 27.5 ± 4.6 | <0.0001 |
| BMI-SDS | 0.4 ± 1.4 | -0.2 ± 0.8 | 2.0 ± 1.2 | <0.0001 |
| Height (cm) | 152.5 ± 13.7 | 149.3 ± 13.2 | 160.1 ± 11.9 | <0.0001 |
| Height-SDS | 0.3 ± 1.0 | 0.2 ± 1.0 | 0.8 ± 0.9 | <0.0001 |
| Weight (Kg) | 51.0 ± 19.1 | 42.8 ± 13.0 | 71.0 ± 16.8 | <0.0001 |
| Weight-SDS | 0.5 ± 1.4 | -0.1 ± 0.8 | 2.1 ± 1.1 | <0.0001 |
| Waist (cm) | 73.4 ± 14.0 | 67.1 ± 9.6 | 88.8 ± 10.6 | <0.0001 |
| SBP (mmHg) | 109.9 ± 13.0 | 106.2 ± 11.6 | 118.6 ± 12.1 | <0.0001 |
| DBP (mmHg) | 60.4 ± 7.9 | 59.2 ± 7.2 | 63.3 ± 8.7 | <0.0001 |
| Hypertension (%) | 3 | 2 | 4 | Ns |
| Glucose (mg/dl) | 86.8 ± 6.6 | 86.8 ± 6.6 | 86.7 ± 6.8 | Ns |
| Creatinine (mg/dl) | 0.60 ± 0.11 | 0.58 ± 0.10 | 0.65 ± 0.11 | <0.0001 |
| Insulin (uU/ml) | 8.8 ± 6.3 | 6.9 ± 4.2 | 13.4 ± 7.8 | <0.0001 |
| HOMA-IR | 1.9 ± 1.4 | 1.4 ± 0.9 | 2.9 ± 1.7 | <0.0001 |
| HDL-cholesterol (mg/dl) | 57.4 ± 15.0 | 60.6 ± 14.9 | 49.2 ± 11.7 | <0.0001 |
| LDL-cholesterol (mg/dl) | 81.9 ± 23.2 | 79.4 ± 22.4 | 85.9 ± 22.7 | 0.04 |
| Total cholesterol (mg/dl) | 157.7 ± 29.0 | 159.7 ± 30.7 | 153.0 ± 24.0 | 0.04 |
| Triacylglycerol (mg/dl) | 64.6 ± 31.0 | 57.2 ± 22.2 | 82.2 ± 40.7 | <0.0001 |
| eGFR (ml/min/1.73m^2^) | 100.7 ± 15.9 | 101.8 ± 16.2 | 97.9 ± 14.8 | 0.04 |
| Renal length (cm) | 9.8 ± 1.0 | 9.5 ± 0.9 | 10.5 ± 1.1 | <0.0001 |
| Renal volume (cm^3^) | 101.6 ± 36.6 | 91.8 ± 30.6 | 126.7 ± 39.9 | <0.0001 |
| Data is presented as mean ± standard deviation (SD), or n (%). BMI: body mass index, SBP: systolic blood pressure, DBP: diastolic blood pressure, eGFR: estimated glomerular filtration rate, HOMA-IR: homeostatic model assessment of insulin resistance, NS: non-significant. P-value<0.05 comparing normal weight vs overweight. Continuous variables are compared with the t-test and proportions are compared with the chi-square. | | | | |
